# Landscape of protein domain interactome

**DOI:** 10.1007/s13238-015-0158-0

**Published:** 2015-05-12

**Authors:** Ting Zhang, Shuang Li, Wei Zuo

**Affiliations:** Shanghai Pulmonary Hospital, School of Medicine, Tongji University, Shanghai, 200092 China; Genome Institute of Singapore, A*STAR, Singapore, 138672 Singapore; Tianjin International Joint Academy of Biomedicine, Tianjin, 300457 China

**Dear Editor,**

Protein domain is usually defined as distinct, compact and stable protein structural unit that folds independently of other such units (Koonin et al., 2002). The majority of proteins, especially in higher organisms, contain multiple domains (Chothia, 1992). Domain view of protein evolution provides many insights into the evolution of pathways and networks, as well as into the general direction of evolution of higher organisms. Domain structure of proteins is also important in understanding protein-protein interactions, as proteins interact with each other not as complete units, but rather via their component domains. Therefore, a protein-protein interaction (PPI) network can be viewed, at higher resolution, as the domain-domain interaction (DDI) network. However the network study in the past decade was still limited to protein but barely zoomed into domain resolution. Although some DDI-based database has been established and analyzed (Stein et al., 2005; Yellaboina et al., 2011), it remains unclear that how different domains play distinct roles in the interaction network and how do they coordinate with each other functionally.

To study the domain-domain interaction (DDI) network, first we established a baseline human protein-protein interaction network composed of 19,139 individual PPIs by integrating several protein-protein interaction databases. We then mapped domains into the PPI network based on Pfam definitions (Sonnhammer et al., 1998). We used information from three databases: iPfam (Finn et al., 2005), 3did (Stein et al., 2005) and DOMINE (Yellaboina et al., 2011) to predict DDIs based on both protein sequence and protein interaction information. In total we identified 46,712 DDIs in our network (Fig. 1A), which include three types of DDIs based on the property of interaction: 1) neighboring intra-DDI, the interactions between neighboring domains within one protein result from their proximity along the amino acid chain; 2) general intra-DDI, the physical interactions between domains within one protein that do not result from their proximity along the amino acid chain but from the folding in 3D space; 3) inter-DDI, physical interactions between domains in interacting proteins (Fig. 1A).

**Figure 1:**
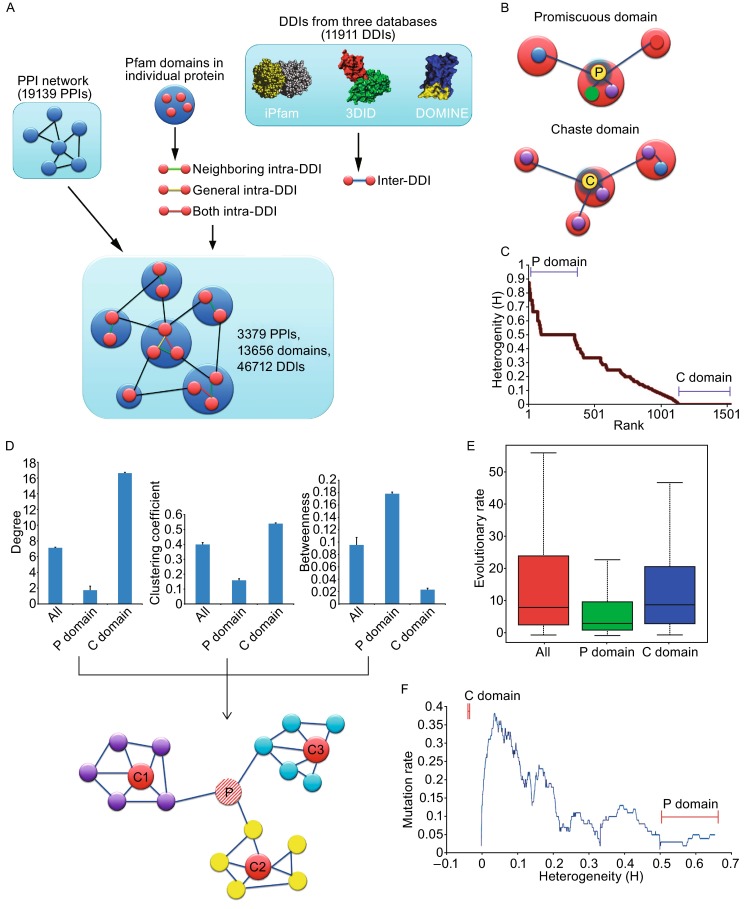
DDI network, domain promiscuity and characteristics of P and C domains. (A) Flowchart of constructing DDI network. (B) Definition of Promiscuous domains (P domains) and Monogamous domains (C domains). The small circles indicate identical (same color) or different (various colors) domains, the large circles indicate proteins harboring single or multiple domains. (C) Identifying P and C domains in DDI network by calculating interaction heterogeneity. (D) Network characteristics of all, P and C domains. Upper panel, the degree, clustering coefficient and betweeness of all, P and C domains. Error bar indicates standard error of the mean. Lower panel, a model of P and C domain localization in network. ‘P’ indicated P domain, and ‘C1/C2/C3’ represented C domain. (E) Box plots of evolutionary rates of all, P and C domains. (F) Mutation distribution on domains of different heterogeneity. The P domains and C domains corresponded to the domains with heterogeneity ≥0.5 and <0.005 respectively

Next we analyzed the topological characteristics of DDI network. Similar to proteins in the PPI network, node degree (k) distribution of domains approximately follow power-law distribution (Fig. S1), which shows that most of the domains are linked to only few other domains. In contrast, some domains such as the “SH2” domain on Grb2 protein are connected to many other domains (k = 122), which is consistent with its central role in dynamic regulation of tyrosine kinase signal, the key signal of eukaryotic cell growth (Tinti et al., 2013). Like the PPI network, the DDI network is also a scale-free network but it has significantly higher betweenness and clustering coefficient than PPI network (Fig. S2). In the DDI network, each domain is represented by multiple nodes (appears more than once) as a portion of different proteins, and each time when it appears, it may have distinct partners. This is unlike in PPI network that each protein is represented by a unique node. So in the DDI network, certain domains may show a tendency to interact with many different types of domains and can be considered as “promiscuous”, or “P domain”. For example, “MAM” domain has 10 interacting partners in the human DDI network, and 9 of them were different domains. This is consistent with previous report that “MAM” domain exist in many functionally diverse proteins to play different roles (Beckmann and Bork, 1993). In contrast, some domains in DDI network tend to participate in limited types (under extreme condition, only one type) of DDIs and therefore can be considered as “chaste”, or “C domain”. For instance, the “Beta-catenin-interacting protein ICAT” domain has 8 partners in DDI network and all of them are the same domain type (“Armadillo repeats”), as the ICAT domain only exist in ICAT protein whose main function is to inhibit beta-catenin/TCF pathway (Graham et al., 2002). The difference between promiscuous and chaste domains is illustrated in Fig. 1B.

To define domain properties quantitatively, for each domain, we counted the types of domains it interacts with to calculate an interacting heterogeneity coefficient H (see the Methods section for the exact definition). The average value of H is equal to 0.16 and whole distribution is shown in Fig. 1C. According to the distribution, we define the domains with H > 0.5 as promiscuous domains (P domains), and those with more than one interacting partner and H < 0.005 as chaste domains (C domains). In total there are 342 P domains and 406 C domains defined, with the other 1448 domains sharing intermediate features of C and P domains. The P domains and C domains were found evenly distributed in intra- and inter-protein DDIs.

We further analyzed the node degrees of P and C domains, as shown in Fig. 1D, we found that node degree for the P domains are lower than average (*P* < 10^−4^ by a Wilcoxon rank-sum test), while C domains’ are higher (*P* < 10^−4^ by a Wilcoxon rank-sum test). About 30% of C domains have degree k ≥ 10. Similarly, for clustering coefficient, which measures the density of network module, P domains are lower than average, while C domains are higher. Therefore, the highly interacted C domains are “hubs” in the network, which function to organize the local network modules. Interestingly, the betweenness of domains, which measures the number of shortest paths between any domain pair that involves a given domain (Yu et al., 2007), is higher than average in the P domains and lower in the C domains. Therefore, P domains are non-hub “bottlenecks” of the network, which usually link different function modules together (Fig. 1D). These results are consistent with Gene Ontology-based function analysis using Pfam2Go, which showed that P domains were enriched in GO terms associated with very general biological functions, such as “metabolic process”, “DNA-directed RNA polymerase activity” and “nucleotidyltransferase activity” (*P*-value < 10^−6^). In contrast, no GO terms were found to be enriched among C domains, suggesting that each C domain may have unique, non-overlapping functions.

Some previous studies (Zmasek and Godzik, 2011) analyzed the evolution pattern of domain repertoire in eukaryotes. Here we examined whether the interacting patterns of domains could affect their evolution. We found no difference in terms of evolutionary rate between C domain and all other domains. However, the evolutionary rate of P domains was much lower than the average (Fig. 1E, *P* < 10^−4^, Wilcoxon rank-sum test) and this effect still exists even if the difference in the contact degree was taken into effect. So the result suggests that the evolution of P domains was constrained by the diversity of their interaction partners.

To identify possibly different roles of P and C domains in diseases, we investigated the distribution of oncogenic mutations in the DDI network. Previous reports (Wang et al., 2012) showed that disease-related mutations tend to be localized in domains linking to another protein (thereafter called “interface” domains). Here we examined the relationship between H and mutation rate, and found that P domains and C domains do not have advantage to accumulate mutations. Instead, the domains with intermediate H values (0.02~0.5) tend to accumulate mutations (Fig. 1F, *P* value < 10^−4^ by a Wilcoxon rank-sum test). Considering that C domains and P domains are hubs and bottlenecks of the DDI network respectively, this observation suggests that oncogenic mutations tend to avoid the topologically important nodes of the biological networks, probably because such mutations in key domains would lead to immediate breakdown of the whole system so become highly deleterious for cancer cell survival.

After analyzing the P and C domains, we continued to study the pattern of DDI pairs. As each domain can appear more than once in the network, each domain pair can also appear more than once. We categorized the 46,712 DDIs into 3,445 pairs, and calculated how often they show up in the network. And we named domain pairs that appear more (or less) frequently than average as “frequent DDIs” (or “rare DDIs”). The most frequent and rare DDIs are listed in Table S1. Within the list, we noticed that the domains in frequent DDIs were functionally similar to each other; instead, domains in rare DDIs usually have different (or complementary) functions. This observation is expected as domains with similar functions tend to coordinate with each other to function together. Evolutionary rate calculation also showed that co-evolving domains (measured by Jensen-Shannon Divergence score, JSD* ≤ 0.05) interact with each other more frequently (Fig. 2A), which should be due to the interacting partners are usually subjective to the identical selective pressure. However, network edge attack analysis indicated that the rare DDIs were more important to maintain the network. Loss of rare DDIs rapidly increased the characteristic path length and decreased the size of largest component, indicating the rapid breakdown of the network (Fig. 2B). The result suggests that rare DDIs function by establishing unique links between different functional modules.

**Figure 2:**
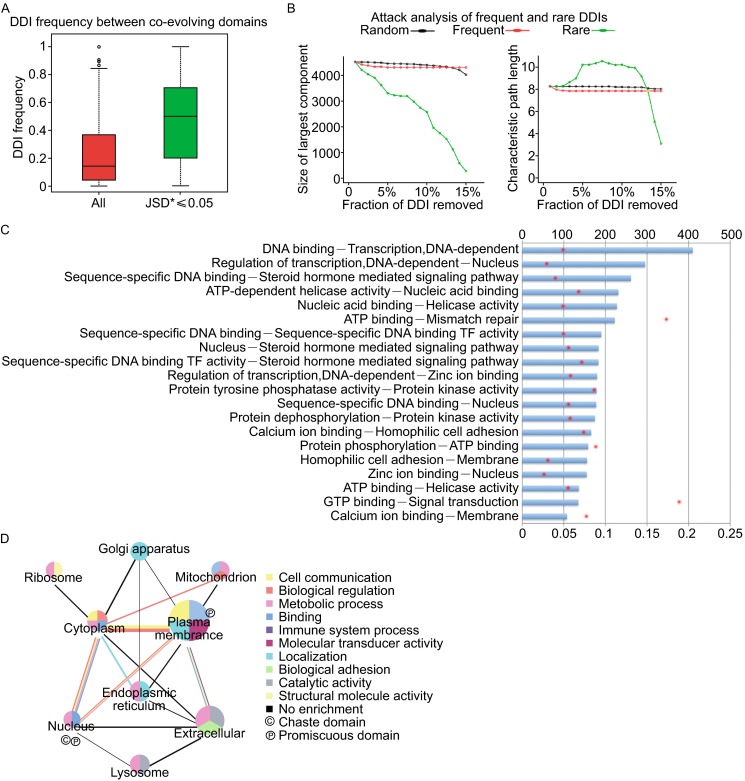
Co-evolution, attack of DDIs, function combination and subcellular localization of DDIs. (A) Box plots of frequency of appearance of all DDI pairs and co-evolving DDI pairs (JSD* < 0.05). (B) Effects of the gradual removal of randomly selected DDI, frequent DDI or rare DDI on the largest component size (upper panel) and characteristic path length (lower panel) of the network. (C) Top 20 most frequent domain function combinations (counting >50). Bars indicated the normalized frequency (ranged from 0 to 1). Red dots indicated the absolute number of the function combinations in the network (ranged from 0 to 400). Redundant combinations were removed. (D) DDIs distribution in subcellular components. The size of the nodes indicates the relative number of DDIs that are within a subcellular component. The thickness of the edges indicates the relative number of DDIs that are between two subcellular components. The colors correspond to different GO functional terms. The circled C and P indicate the enrichment of C domain and P domain

To understand the pattern how different biological functions are coordinated through combination of domains, we integrated the domain function information onto the DDI network. We found that there are some function combination appears more frequently than by chance. For example, in the network there are 73 domains annotated with “double-stranded RNA binding” function and 29 domains annotated with “RNA processing” function, and they form 11 function combinations with the frequency (=0.239) much higher than statistically expected (=0.066, *P* < 0.01 by a Wilcoxon rank-sum test). This is also consistent with our knowledge that protein binding of double-stranded viral RNA and processing it are two closely related biological processes. The top 20 frequent function combinations are listed in Fig. 2C.

Furthermore, to understand the spatial distribution of domains, we mapped the subcellular location information of domains to the DDI network. We found that the communication between domains within the following locations is most frequent: “extracellular part-plasma membrane”, “plasma membrane-cytoplasm” and “cytoplasm-nucleus” (Fig. 2D). This is in consistent with our understanding that the “extracellular part-plasma membrane-cytoplasm-nucleus” is the most classical signal transduction axis in cells. Furthermore, we uncovered the complex relationship between domain function and domain subcellular localization. For instance, we showed that “biological adhesion” is a unique function that fulfilled by DDI within extracellular part, and “immune system process” is fulfilled by DDI between extracellular part and plasma membrane. Such observation is also consistent with biological knowledge in prior (Gilbert, 1986; Kupiec-Weglinski et al., 1993). We also found that P domains are enriched in both plasma membrane and nucleus, while C domains are comparatively limited in nucleus. This could be explained as the domains on the cell surface (plasma membrane) need to be promiscuous to adapt to various outside environment. The analysis above altogether indicated that in higher organism, domains are functionally well combined and spatially well organized. Altogether our studies uncover the landscape of how domains interact with each other to make the whole biological system works properly.

## Electronic supplementary material

Supplementary material 1 (XLSX 52 kb)

Supplementary material 2 (TIF 131 kb)

Supplementary material 3 (TIF 1676 kb)
