# Supplementary figures and images for: Landscape of protein domain interactome
Source: Protein Cell. 2015 May 12;6(8):610–4. doi: 10.1007/s13238-015-0158-0 (PMC4506283; doi:10.1007/s13238-015-0158-0)

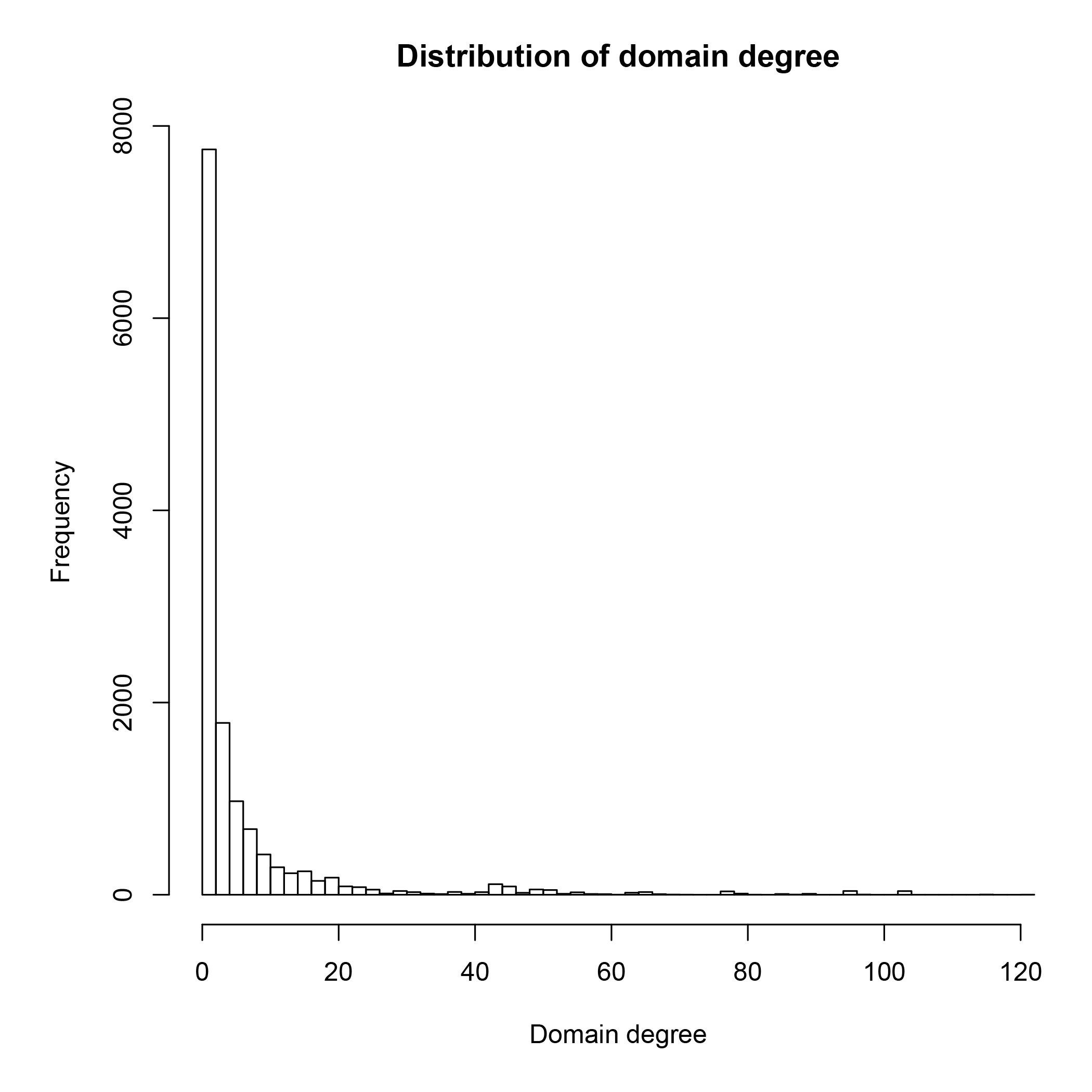

Supplement: Supplementary file 2 — Supplementary material 2 (TIF 131 kb) [file 13238_2015_158_MOESM2_ESM.tif]

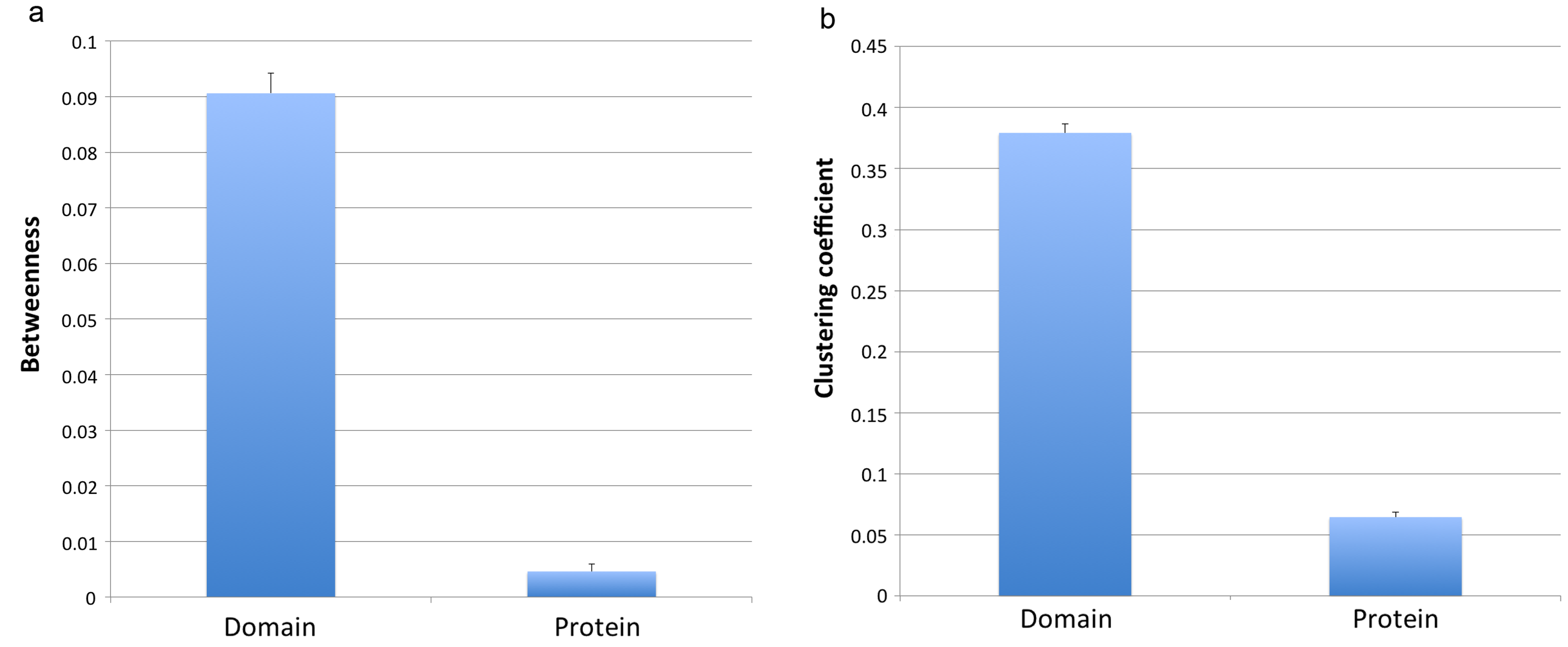

Supplement: Supplementary file 3 — Supplementary material 3 (TIF 1676 kb) [file 13238_2015_158_MOESM3_ESM.tif]
